# Sphingolipids: membrane microdomains in brain development, function and neurological diseases

**DOI:** 10.1098/rsob.170069

**Published:** 2017-05-31

**Authors:** Anne S. B. Olsen, Nils J. Færgeman

**Affiliations:** Villum Center for Bioanalytical Sciences, Department of Biochemistry and Molecular Biology, University of Southern Denmark, 5230 Odense M, Denmark

**Keywords:** sphingolipid, ganglioside, membrane microdomain, raft, brain, neurological disease

## Abstract

Sphingolipids are highly enriched in the nervous system where they are pivotal constituents of the plasma membranes and are important for proper brain development and functions. Sphingolipids are not merely structural elements, but are also recognized as regulators of cellular events by their ability to form microdomains in the plasma membrane. The significance of such compartmentalization spans broadly from being involved in differentiation of neurons and synaptic transmission to neuronal–glial interactions and myelin stability. Thus, perturbations of the sphingolipid metabolism can lead to rearrangements in the plasma membrane, which has been linked to the development of various neurological diseases. Studying microdomains and their functions has for a long time been synonymous with studying the role of cholesterol. However, it is becoming increasingly clear that microdomains are very heterogeneous, which among others can be ascribed to the vast number of sphingolipids. In this review, we discuss the importance of microdomains with emphasis on sphingolipids in brain development and function as well as how disruption of the sphingolipid metabolism (and hence microdomains) contributes to the pathogenesis of several neurological diseases.

## 1. Introduction

The nervous system is among the tissues in the mammalian body that has the highest lipid content as well as the highest lipid complexity. This complexity can be ascribed to the lipid class of sphingolipids. Sphingolipids are particularly abundant in the brain and are essential for the development and maintenance of the functional integrity of the nervous system [1,2]. The grey matter and neurons are highly enriched in the glycosphingolipid (GSL) subgroup gangliosides, while the sphingolipid species sphingomyelin (SM), galactosylceramide (GalCer) and sulfatide are enriched in oligodendrocytes and myelin [3,4]. However, the sphingolipid profile of the brain is far from static as it continuously changes as the brain develops and ages [4–6].

The plasma membrane is a very heterogeneous environment composed of several hundreds of different lipid species [7]. Yet the movement of lipids and proteins has been shown to be more or less restricted due compartmentalization of the membrane as a consequence of lipid–lipid, lipid–protein and membrane–cytoskeletal interactions [8]. The compartmentalization is a consequence of the generation of microdomains that can be described as dynamic assemblies enriched in cholesterol and/or sphingolipids, which are located in the outer leaflet of the plasma membrane [9]. The saturated acyl chains of the sphingolipids allow these to pack more readily against cholesterol, which leads to the formation of highly packed liquid-ordered phases that are distinct from the bulk liquid-disordered phase of the plasma membrane [10]. Indeed, the plasma membrane of cells in the nervous system is highly enriched in both cholesterol and sphingolipids, especially GSLs [11–13]. The existence of microdomains has been highly debated, as they have proven difficult to define experimentally and thus study. Recent studies indicate that this may very well be attributed to the heterogeneity of microdomain composition, which is reflected in the numerous combinations of lipids as well as proteins [14]. Morphologically only one type of microdomain has been defined, namely the caveolar microdomain. Caveolae are small 50–100 nm invaginations of the plasma membrane where the protein caveolin associates with membrane enriched in cholesterol and sphingolipids [15]. However, sphingolipid- and cholesterol-dependent microdomains with a diameter less than 20 nm and an average lifespan of 10–20 ms have been identified in living cells [16,17].

Neurons and oligodendrocytes are highly polarized cells, and compartmentalization of signalling events is required in order to maintain normal neuronal physiology, including neuronal differentiation, polarization, synapse formation, synaptic transmission and glial–neural interactions [18]. Studies show the involvement of sphingolipids in all these processes (reviewed in [2,3,18,19]). Dysregulation of the sphingolipid metabolism has been associated with a vast number of neurological diseases via disturbances of membrane organization [2,20,21]. The list of ion channels and signalling receptors that localize to and are regulated by sphingolipid microdomains in the brain is expanding, but for a long time cholesterol has been the pivot when studying the formation of membrane microdomains. In the present review, we discuss the connection between sphingolipids and their involvement in membrane microdomains, brain development as well as neurological diseases.

## 2. Biosynthesis and metabolism of sphingolipids

Numerous studies during the past decades have led to significant advances in our understanding of the biosynthesis and degradation of the sphingolipid pathway [22–24]. Ceramide constitutes the basal building block for the more complex sphingolipids and consists of a long-chain sphingoid base (LCB), sphinganine or sphingosine, with a fatty acid attached via an amide bond at the C2 position [25]. More complex sphingolipids are generated by attaching various head groups in the C1 position of ceramide [26]. Sphingolipids constitute a very diverse group of lipids, which counts several hundred different species. The vast number of species originates from the structural diversity and combinations within LCBs, fatty acids and head group variants [27–30].

**Figure 1.**
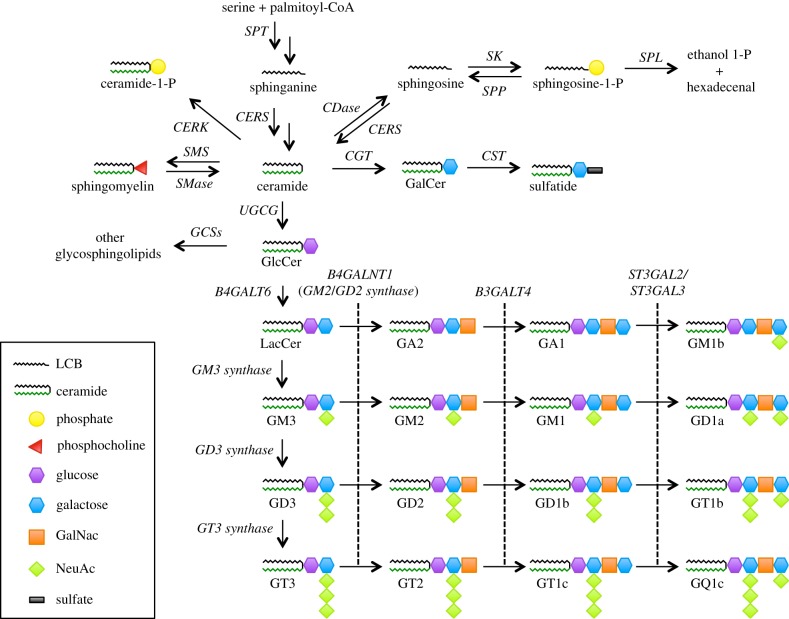
Overview of the sphingolipid metabolism. Sphingolipids encompass a broad spectrum of lipids. Ceramide is central in the sphingolipid metabolism as it serves as a precursor for the synthesis of more complex sphingolipids. Ceramide is synthesized de novo from serine and palmitoyl-CoA. Subsequently, complex sphingolipids are synthesized by attachment of different head groups to ceramide as indicated in the figure. In particular, ganglioside biosynthesis has been highlighted. Gangliosides are mono- or multi-sialosylated glycosphingolipids, which are highly abundant in the nervous system. Their synthesis is a multistep process of addition of sugars and sialic acids. Degradation of complex sphingolipids contributes to the pool of ceramide that can either be re-used for complex sphingolipid synthesis or alternatively be broken down. Degradation of glycosphingolipids by glycosidases and sialidases is not indicated in the figure. Abbreviations: beta-1,4-N-acetyl-galactosaminyl transferase 1 (B4GALNT1), beta-1,3-galactosyltransferase 4 (B3GALT4), beta-1,4-galactosyltransferase 6 (B4GALT6), ceramidase (CDase), ceramide galactosyltransferase (CGT), ceramide kinase (CERK), ceramide synthase (CERS), galactosylceramide sulfotransferase (CST), glycosphingolipid synthases (GCSs), serine palmitoyltransferase (SPT), sphingomyelin synthase (SMS), sphingomyelinase (SMase), sphingosine kinase (SK), sphingosine 1-phosphate phosphatase (SPP), sphingosine 1-phosphate lyase (SPL), ST3 beta-galactoside alpha-2,3-sialyltransferase 2 (ST3GAL2), ST3 beta-galactoside alpha-2,3-sialyltransferase 3 (ST3GAL3).

Figure 1 outlines the synthesis and major parts of the metabolism of sphingolipids. The de novo synthesis of ceramide is initiated at the cytosolic leaflet of the endoplasmic reticulum (ER) where it is generated in a four-step process [31–33]. Briefly, serine and palmitoyl-CoA are condensed to 3-ketodihydrosphingosine by the serine palmitoyltransferase (SPT). 3-ketodihydrosphingosine is rapidly reduced to sphinganine before a ceramide synthase (CERS) converts sphinganine to dihydroceramide. Lastly, dihydroceramide is desaturated resulting in the formation of ceramide [34]. Six different mammalian CERSs have been identified. They all display unique expression profiles as well as fatty acyl-CoA specificity ranging from C14 to C26 carbon atoms [35]. For instance CERS1, which mainly uses C18 CoAs, is highly expressed in the brain and skeletal muscles, while CERS2 is more ubiquitously expressed, but with a high expression in oligodendrocytes and generates mainly C20–C26 ceramides.

Once formed, ceramide can be converted into more complex sphingolipids through different pathways. In the ER lumen, ceramide can either be turned into ceramide phosphoethanolamine or be glycosylated to GalCer [36,37]. GalCer is a precursor for sulfatides that along with GalCer are important components in myelin that insulates neurons in the central nervous system (CNS) [22]. Ceramide can also be delivered to the Golgi apparatus where it is converted into SM or glucosylceramide (GluCer). GluCer can then be converted into lactosylceramide (LacCer) by addition of galactose [22]. LacCer serves as an intermediate in the synthesis of more complex GSLs, which is conducted by sequential transfer of sugars and other chemical groups by galactosyltransferases, sialyltransferases, N-acetylgalactosamine transferases and GalCer sulfotransferases all residing in the Golgi apparatus [24]. Gangliosides constitute a rather large GSLs subgroup, which is particularly abundant in the grey matter of the brain. Combinations of glucose, galactose and N-acetylgalactosamine constitute the head groups of gangliosides and give rise to a highly structural diversity [38].

Once synthesis is complete, SM and GSLs are relocated to the plasma membrane where they are known to participate in microdomain formation [24]. The fact that complex GSL synthesis occurs on the luminal side of Golgi apparatus renders that GSLs are oriented towards the extracellular matrix after trafficking to the plasma membrane. The plasma membrane is very dynamic in the sense that microdomains form and disperse in response to cellular signals. Sphingolipids in the plasma membrane can undergo remodelling, which allows for fast modulation of membrane composition in response to stimuli. For instance, ceramide can be generated from both SM and GM3 by the action of sphingomyelinases (SMases) and N-acetyl-α-neuraminidase 3 (Neu3) in combination with glycosylhydrolases, respectively [39,40], and SM can be re-synthesized by the action of SM synthase 2 [41].

Removal of sphingolipids from the plasma membrane occurs through the endolysosomal pathway where SM and GSLs are degraded to ceramide by the action of acid sphingomyelinase (aSMase) and glycosidases, respectively [42]. Here ceramide is further deacylated to sphingosine by the acid ceramidase (aCDase). Sphingosine can either be re-acylated by CERSs, allowing sphingosine to enter the recycling pathway and be used as a precursor for complex sphingolipids, or alternatively be broken down.

As the function of each sphingolipid species depends on their specific structure, pathway and subcellular localization [43], tight regulation of the sphingolipid network is necessary in order to ensure proper brain functions, as discussed below.

## 3. Sphingolipid composition during brain development and ageing

**Figure 2.**
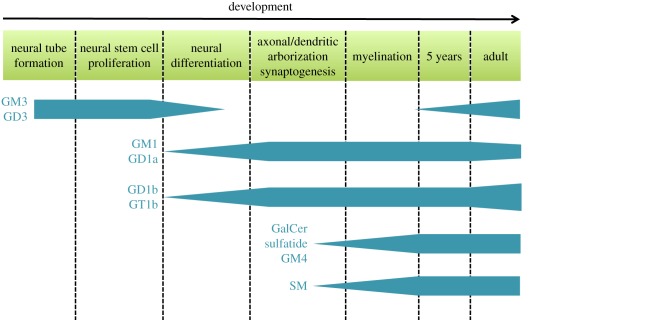
Outline of how key sphingolipids change during neurodevelopment and ageing. During development of the nervous system the ganglioside profile changes from the simple species (GM3 and GD3) early in embryogenesis to the more complex gangliosides (GM1, GD1a, GD1b and GT1b) later in embryogenesis. Concurrent with myelination, the levels of the myelin sphingolipids sphingomyelin (SM), galactosylceramide (GalCer), sulfatide and GM4 increase. In adulthood, the ganglioside profile changes again with increasing levels of GM3, GD3, GD1b and GT1b, while the levels of GM1 and GD1a decrease.

The sphingolipid composition of the human brain has been studied in detail since the 1960s [4,5,44–48]. Numerous studies have shown that sphingolipids are found in high concentrations in nervous system and that the distribution and composition of sphingolipids are distinct in different regions as well as cell types of the CNS. The grey matter and neurons are particularly enriched in gangliosides, while oligodendrocytes and myelin are highly enriched in galactolipids GalCer and its sulfated derivate sulfatide [48]. Furthermore, the sphingolipid profile changes continuously as the brain develops and ages (figure 2), indicating that sphingolipids are involved in the differentiation and maintenance of neural functions [4–6]. Consistently, expression of enzymes involved in sphingolipid biosynthesis follows brain development [3].

Gangliosides are major components of the neuronal membranes as they account for 10–12% of the lipid content [49]. They are located on the external leaflet of the plasma membrane from where they participate in key processes maintaining neuronal functions such as neuronal development and myelin stability [13,38,49]. In the adult mammalian brain, the four major brain gangliosides are GM1, GD1a, GD1b and GT1b [46,50]. It is well known that the ganglioside profile changes remarkably during development of the nervous system as well as throughout life, and these changes are region-specific [46,51]. The tight regulation of ganglioside expression is thought to instruct brain maturation processes, which as the brain ages are being reversed [52]. The importance of the ganglioside changes in brain maturation is highlighted by the fact that they correlate with several neurodevelopmental milestones including neural tube formation, neuronal differentiation, axongenesis, outgrowth of dendrites and synaptogenesis. During embryogenesis in mice, there is a marked shift from the simplest gangliosides, GM3 and GD3, to the more complex gangliosides [53]. There is a rapid increase in GD1a in human cortical layers between weeks 16 and 30 of gestation, coinciding with a rapid cortical synaptogenesis [51]. Increase of GM1 and GD1a in the human frontal cortex correlates with neuronal differentiation, outgrowth of dendrites and axons, as well as synaptogenesis [46]. Furthermore, the four major brain gangliosides GM1, GD1a, GD1b and GT1b all increase significantly from 5 months of gestation to 5 years of age, which is coinciding with the most active period of myelination [46]. After 5 years of age, the proportion of GM1 and GD1a decreases, while the levels of GM3, GD3, GT1b and GD1b increase with age [6,46]. It is not only the ganglioside head group that changes with age. The length of LCB and the fatty acid attached to the LCB also changes [45]. The most common ganglioside chain lengths of both LCBs and fatty acids in the human brain are C18, but C20 species increase from birth [30,45,49].

SM and the galactolipids are major lipids in myelin and their concentrations increase proportionally during the development of myelin [44]. GalCer and sulfatide comprise 23 wt% and 4 wt% of the total lipid of myelin, respectively [54]. During the first 2 years of post-natal life, there is a marked shift in the type of SM in the white matter [5]. C18 SM decreases from 82% to 33%, while C24:0 SM and C24:1 SM increases from 4% to 33% and 2% to 11%, respectively. This pronounced shift from medium-long-chain to very-long-chain SMs is not observed in the cerebral cortex. Here, the SM pattern remains fairly constant from birth to 2 years of age with C18:0 SM constituting more than 85% [5]. GalCer in myelin is enriched in very-long-chain fatty acids (C22–C26) [55]. Thus, overall the dominating fatty acid in ceramide found in the grey matter of the brain is C18, while C24 dominates the white matter. This is in line with a high expression of CERS1 and CERS2 in the grey and white matter, respectively.

It is important to keep in mind that the changes in sphingolipid composition can be highly regional. For instance, there is an age-dependent increase in SM and GM1 in synaptosomes isolated from mice brains [56,57]. Enrichment of GM1 occurs in microdomains isolated from synaptosomes that are resistant to cholesterol depletion indicating the presence of GSL microdomains at synaptic terminals [57]. Thus local changes in the sphingolipid profile, which might be hidden in the overall level of brain sphingolipids, can be functionally important.

## 4. Microdomains in brain development and maintenance

**Figure 3.**
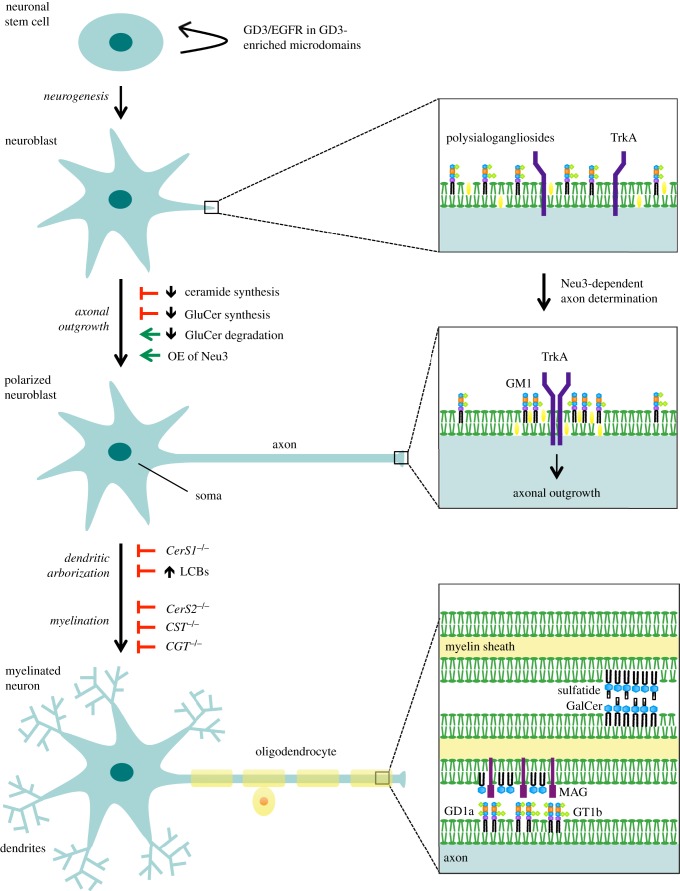
Roles of sphingolipids in neuronal and glial development and interaction. Sphingolipids are involved in multiple steps of the development of the nervous system. Ganglioside GD3 is important for neuronal stem cell proliferation during which it is found co-localizing with the epidermal growth factor receptor (EGFR) in microdomains. Inhibition of ceramide and glucosylceramide (GluCer) synthesis both inhibit axonal outgrowth, while inhibition of GluCer degradation and overexpression (OE) of the sialidase Neu3 stimulate axonal outgrowth. Neu3 stimulates breakdown of polysialogangliosides to GM1, which recruits the nerve growth factor receptor TrkA into microdomains thereby promoting axonal outgrowth. Dendritic arborization is reduced in *CerS1^−/^*^−^ mice, which is likely to be caused by increase of long-chain bases (LCBs). Myelination defects have been found in mice deficient in ceramide synthase 2 (CERS2), GalCer sulfotransferase (CST) as well as UDP-galactose:ceramide galactosyltransferase (CGT). Sphingolipids are important for myelin stability. Galactosylceramide (GalCer) and sulfatide in microdomains in opposing membranes of the myelin sheath form glycosynapses important for long-term myelin stability. GD1a and GT1b in axonal membrane microdomains contribute to myelin stability by interacting with myelin-associated glycoprotein (MAG) residing in the myelin sheath.

Neurons and oligodendrocytes are highly polarized cells with morphological differences that allow them to carry out specialized functions. This is attributed to the organization of their membranes in specific sub compartments of which sphingolipids play an important role. During neuronal development, the composition and organization of synaptic membranes are being remodelled. Establishment and maintenance of membrane organization is crucial in order to maintain neuronal physiology including neuronal differentiation, polarization, synapse formation and glial–neural interactions. Hence, perturbations of the sphingolipid network, and thus membrane microdomains, have been implicated in multiple dysfunctions affecting neuronal physiology. The diverse roles of sphingolipids in brain development and maintenance are described below and outlined in figure 3.

### 4.1. Neural differentiation, polarization and synapse formation

As discussed above, the ganglioside profile changes during embryonic development with simple gangliosides dominating the early phases. The simple ganglioside GD3 may have a central role in early neurogenesis as the activity of the GD3 synthase increases during this period, where it also constitutes the major ganglioside [58]. This is supported by studies showing GD3 being crucial for sustaining the self-renewal capability and neurogenesis of mice neural stem cells [59,60]. Sorting of the epidermal growth factor receptor (EGFR) has proven to be essential for the regulation of stem cell renewal, and it has been shown that EGFR co-localizes with GD3 in membrane microdomains in mice neural stem cells [59]. Furthermore, neural stem cells from GD3 synthase-deficient mice have reduced level of EGFR expression and accelerated EGF-induced EGFR degradation consistent with decreased self-renewal capacity [59].

Early studies have shown that exogenously supplemented gangliosides promote neurite outgrowth in neuroblastoma cell lines, primary neurons and sensory ganglia [61–64]. The nerve growth factor (NGF) induces neurite extension through binding to and activation of the tropomyosin receptor kinase A (TrkA) receptor leading to the activation of the Ras/Raf/MEK/Erk pathway [65]. Accordingly, exogenous GM1 binds to the TrkA receptor in membrane microdomains augmenting NGF-induced activation in the rat pheochromocytoma cell line PC12 and in rat primary hippocampal neurons [66–69]. Interestingly, overexpression of the GD3 synthase in PC12 cells leads to continuous activation of TrkA signalling through the Ras/Raf/MEK/Erk pathway [70]. Overexpression of the GD3 synthase also leads to an increase in GD1b and GT1b, while the level of GM1 decreases indicating that GD1b and GT1b might also be involved in regulating TrkA signalling. Surprisingly, overexpression of the GM1 synthase (B3GALT4) in PC12 cells prevents NGF-induced activation of TrkA, which is probably due to significant changes in the intracellular localization of the receptor [71]. Thus, balancing the level of GM1, as well as GD1b and GT1b, is important in controlling the TrkA signalling response in neuronal polarization.

Axonal outgrowth, projection of the axon from the soma of a neuron towards a target cell, is an essential process in the wiring of the neural network. It has been shown that inhibition of CERS activity leading to depletion of ceramide, SM and GSLs significantly reduces axonal outgrowth in cultured hippocampal neurons [72]. The depletion of GSLs might be the primary effector responsible for this phenotype as inhibition of GluCer synthesis decreases axonal outgrowth as well as axonal branching in cultured hippocampal neurons [73], whereas the opposite effect is observed when GluCer degradation is inhibited. As inhibition of ceramide synthesis leads to build-up of the ceramide precursors, sphingosine and sphinganine, it is possible that these precursors contribute to the decrease in axonal growth as treating distal neuritis of cultured rat sympathetic neurons with exogenous sphingosine causes neurites to retract and/or degenerate [74]. GM1 may very well be a central player in determining axonal fate, as Neu3, which converts more complex gangliosides to GM1, is essential for determining which growth cone of unpolarized neurons will become the axon (axon specification) in rat primary embryonic hippocampal neurons [69]. Consistently, overexpression of Neu3 accelerates the axon specification as well as axonal growth, while suppressing Neu3 activity blocks axonal generation [69]. Furthermore, NGF-induced polarization is significantly enhanced by Neu3 overexpression, which is in line with a pronounced increase in TrkA phosphorylation, indicating Neu3 induces axon specification through enhancing TrkA signalling [69].

Purkinje cells are some of the largest neurons in the human brain and are characterized by their extensive dendritic arborization. It has been shown that inhibition of CERS activity compromises dendrite genesis by decreasing length, expanse and arborization of dendrites along with reduced survival of rat Purkinje cells [75]. Consistent with the fact that CERS1 is the primary neuronal CERS, loss of CERS1 function in mice leads to shortening of dendritic arbours and degeneration of Purkinje cells [76]. Similar phenotypes have been observed for inhibition of SPT in Purkinje neurons, indicating that the de novo sphingolipid synthesis is pivotal for dendritic development and survival [77]. Pinpointing the sphingolipid species responsible for these phenotypes is highly challenging. Purkinje cell-specific knockout (KO) of the glucosyltransferase has little effect on dendrites, but leads to axonal degeneration and disrupted myelin sheath, which suggests that GSLs are not responsible for the dendritic phenotypes [78]. Inhibition of CERS activity and loss of CERS1 in Purkinje cells result in accumulation of the ceramide precursors sphinganine, sphingosine and 1-deoxy-sphinganine [76,79]. Ectopic expression of CERS2 in neurons suppresses Purkinje cell death in *CerS1^−/−^* mice through restoration of LCBs to wild-type levels indicating that elevation of LCBs is the primary cause of neuronal death in CERS1-deficient mice [79]. This is supported by the observation that treatment of cultured neurons with LCB levels corresponding to the levels found in the brain of *CerS1^−/^*^−^ mice causes neurite fragmentation [79]. Thus, LCBs may be a central player of neurodegeneration upon disruption of the sphingolipid metabolism.

During brain development neurons migrate to a final localization where they interact with their appropriate signalling partners ensuring correct formation of pre- and post-synaptic elements at the right time and place. Early in the developing rat nervous system the expression of a variant of GD3, 9-O-acetyl GD3, appears to be involved in glial-guided neuronal migration and neurite outgrowth [80]. A similar role might be performed by GM1 in the early stages of the human brain development as GM1 has been implicated in glial-neuronal contacts during the migration of neuroblasts [81].

Synapses are key sites of communication between neuronal cells where the presynaptic cell propagates a response to the postsynaptic cell through either a chemical or electrical signal. Compartmentalization is pivotal at synapses in order to transmit the signal as efficiently as possible. In rat hippocampal neurons disruption of microdomains by simultaneous cholesterol depletion and CERS inhibition leads to fewer, but larger clusters of both the excitatory AMPA receptor and the inhibitory GABA_A_ receptor [82]. Structurally the microdomain disruption means loss of inhibitory and excitatory synapses as well as reduction in the number of dendritic spines [82]. As excitatory synapses are usually located on spines in hippocampal neurons the morphological consequences caused by microdomain disruption most probably also have functional consequences. This is in line with the fact that gradual loss of synapses and spines are characteristic for neurodegenerative diseases [83].

### 4.2. Sphingolipids mediating axon-glial architecture

Myelination of axons is crucial in order to provide electrical insulation of axons ensuring rapid and efficient action potential propagation. Proper myelination in the CNS requires oligodendrocytes to form multilayered myelin membranes wrapped around axons, the myelin sheaths, which involves precise sorting and compartmentalization of myelin proteins as well as GSLs and galactosphingolipids into microdomains (reviewed in [3,55,84]). Disruption hereof leads to deterioration of myelin, resulting in axon degeneration, which contributes to the pathogenesis of demyelinating diseases [14]. Indeed, myelin defects have been associated with several enzymes of the sphingolipid pathway including the GM2/GD2 synthase, UDP-galactose:ceramide galactosyltransferase (CGT) and GalCer sulfotransferase (CST) [85–89].

Formation and stability of the myelin sheath depends on protein–lipid interaction between the sheath and axon, but it also depends on lipid–lipid interactions between myelin sheath layers. Gangliosides GD1a and GT1b localized in microdomains in the axonal membrane interact and regulate the myelin protein myelin-associated glycoprotein (MAG) [87,90,91], which itself is located in GalCer-enriched microdomains in mature myelin [92]. Disturbance of GD1a and GT1b in neurons by either neuramidase treatment, blockage of ganglioside biosynthesis or blockage of access by specific IgG-class anti-ganglioside antibodies all prevent MAG-mediated inhibition of neurite outgrowth [90]. Other major myelin proteins found within GalCer-enriched microdomains in mature myelin are myelin basic protein (MBP), 2′,3′-cyclic-nucleotide 3′-phosphodiesterase (CNP), myelin/oligodendrocyte glycoprotein (MOG) and proteolipid protein (PLP) [92]. Sorting of the myelin proteins into the GalCer-enriched microdomains may already occur in the Golgi apparatus as it has been shown that PLP association with GalCer- and cholesterol-enriched microdomains in the Golgi is necessary for correct localization in the membrane of oligodendrocytes [93]. Besides controlling the localization of the major myelin proteins, GalCer contributes to the long-term stability of myelin by interacting with sulfatide located in the membrane of opposing layers in the myelin sheath forming what is known as a glycosynapse [94,95].

Between myelin sheaths there are regularly spaced unmyelinated regions of the axon, also known as nodes of Ranvier, where ion channels driving the action potential propagation are highly enriched. The structural stability of the nodes and their neighbouring functional regions (paranodal, juxtaparanodal and internode region) depends on cell adhesion molecules (CAMs) in the axonal and glial membranes, as well as oligodendrial GalCer and sulfatide [85,86,96,97]. Disturbances of the nodes of Ranvier have been observed in CGT-deficient mice lacking the ability to synthesize both GalCer as well as sulfatide, and in CST-deficient mice, which are unable to synthesize sulfatide from GalCer [96,98]. Both mice strains have disrupted axo–glial interactions, which in turn lead to dislocation of axolemma proteins including juxtaparanodal K^+^ channels transcending into the paranodal region and diffuse distribution of the axonal CAMs contactin-associated protein (Caspr) and paranodin [86,96,98]. These disturbances result in conduction deficits and pronounced tremor combined with progressive ataxia [86,89]. Similar ultrastructural dysfunctions may very well be present in the *CerS2^−/−^* mouse. As mentioned, CERS2 is responsible for the synthesis of very-long-chain ceramides including C22 and C24 ceramide. The lipid composition of myelin in CERS2-deficient mice is significantly changed on the level of ceramide, SM, and in particular GalCer [99]. As WT mice age from birth to 1 month, the acyl chain length of GalCer changes from C18 to C22/C24 coinciding with active myelination. As *CerS2*^−/−^ mice are not able to compensate for the loss of C22 and C24 GalCer, these mice develop unstable myelin including degeneration and detachment [99]. This is consistent with myelin degeneration after the age of 1.5 months as seen in CGT-deficient mice [86]. Not only galactolipids have proven to be important for maintaining the structure of the nodes of Ranvier; imbalance in the ganglioside profile has been shown to challenge their stability. Mice deficient in the GM2/GD2 synthase have normal levels of ganglioside, but express only the simple gangliosides GM3 and GD3, yet no major abnormalities have been observed in the gross development of their nervous system [100]. However, ultrastructural defects have been detected, including axon degeneration and demyelination resulting in progressive behavioural neuropathies as deficits in strength, coordination and balance as well as development of tremor and catalepsy [88,100]. GM2/GD2 synthase-deficient mice have abnormal microdomain composition at the nodes of Ranvier affecting the myelination, which might be explained by attenuated expression of the axonal CAM Caspr and the glial CAM neurofascin 155 (NF155) [85]. Furthermore, in these mice the microdomain disturbance leads to mislocalization of K^+^ channels and Na^+^ channels that in turn results in ion channel dysfunction and reduced motor nerve conduction [85]. These effects only get more prominent with age.

### 4.3. Neuronal plasticity

The brain is far from being static after development has completed. Throughout life the brain adapts to stimuli, which underlie functions of learning, behaviour and memory, and it is this ability that helps the brain to overcome brain damage. Neuronal plasticity is evident as modulation of synapse efficacy, which is controlled by organization and composition of the synapse structure. As sphingolipids play an important role in organizing neuronal membranes, it is not surprising that alterations in the sphingolipid pathway have been associated with disturbances in neuronal plasticity.

Several lines of evidence point towards the neutral sphingomyelinase-2 (nSMase) being able to modulate postsynaptic function. nSMase is enriched in the hippocampus, where it quickly can hydrolyse SM to ceramide [101]. It has been shown that nSMase regulates excitatory postsynaptic currents by controlling membrane insertion and clustering of NMDA receptors [102]. Not surprisingly, mice deficient in nSMase show compromised plasticity by having impaired spatial and episodic-like memory [103]. It is becoming evident that the balance between SM and ceramide is important in order to maintain a normal state of mind as increased level of ceramide has been associated with major depression [104,105]. Several anti-depressant drugs have been shown to inhibit the aSMase thereby lowering the concentration of ceramide in the hippocampus resulting in increased neuronal proliferation, maturation and survival as well as improving stress-induced depression in mice [104]. Similar effects are seen in mice deficient in aSMase activity, while the reverse is observed in mice accumulating ceramide by either overexpression of aSMase, heterozygous loss of acid ceramidase, pharmalogic inhibition of ceramide metabolism or direct injections of C16 ceramide into the hippocampus [104]. Thus the concentration of ceramide appears to determine the behaviour mediated through hippocampal functions.

Synaptic plasticity covers several phenomena including long-term potentiation (LTP), the strengthening of synapse signalling through repeated presynaptic stimulation. LTP is one of the major mechanisms constituting the basis for memory and learning. The molecular mechanisms governing LTP are diverse, and are neuronal and region-specific [106]. In the hippocampus, regulation of the glutamate receptor NMDA in number and localization in postsynaptic membranes is one of these mechanisms. NMDA receptors localize to membrane microdomains enriched in sphingolipids [102,107], indicating that the sphingolipids may very well be involved in NMDA-mediated LTP. Indeed, several studies have associated exogenous gangliosides with regulation of LTP in hippocampal neurons [108,109]. Both exogenous GQ1b and stimulation of ganglioside synthesis enhance ATP-induced LTP in hippocampal CA1 neurons, which can be blocked by NMDA antagonists [109]. Furthermore, GQ1b has been found to increase brain-derived neurotrophic factor (BDNF), an important protein in synaptic plasticity, through regulation of the NMDA receptor in rat cortical neurons [110]. Understanding the mechanisms behind sphingolipid modulation of neural plasticity will be a valuable tool in treatment of disabilities of learning, behaviour and memory as well as brain injury.

## 5. Brain ion channels and receptors in microdomains

A vast number of ion channels and receptors have been reported to localize to brain membrane microdomains (reviewed in [7] and [111]). However, the focus has primarily been on microdomains defined by detergent methods and cholesterol depletion and less on the role of sphingolipids. Table 1 gives an overview of neuronal ion channels and receptors that have been shown to be affected by sphingolipids. When interpreting the effect of changed sphingolipid metabolism on ion channel/receptor function, it should be kept in mind that table 1 includes findings in neuronal cells as well as in non-neuronal model cells. Future research will help determine whether or not the findings in the non-neuronal cells can be equated with neurons.

**Table 1.** Examples of neuronal ion channels and receptors being affected by sphingolipid metabolism.

	tissue/cell line	functional effects/comments	references
***ion channels***			
α3β2 nicotinic acetylcholine receptor	rat hippocampal neurons	removal of cholesterol and hydrolysis of SM into ceramide decreases desensitization half-time	[112]
α7 nicotinic acetylcholine receptor	rat hippocampal neurons	removal of cholesterol and hydrolysis of SM into ceramide slows down the desensitization kinetics including increased agonist affinity	[112]
Kir1.1	oocytes	hydrolysis of SM into ceramide inhibits K^+^ conductance and decreases ionic and gating currents	[113]
Kv1.3	jurkat T-lymphocytes	constitutively localized in sphingolipid-rich microdomains; generation of ceramide mediates formation of large ceramide-enriched domains and inhibits channel activity	[114]
	oocytes	hydrolysis of SM into ceramide decreases ionic and gating currents	[113]
Kv1.5	Ltk cells	Co-localizes with caveolin; inhibition of CERS activity induces hyperpolarization shift of the activation and inactivation curve	[115]
Kv2.1	oocytes	hydrolysis of SM into ceramide-1-phosphate induces hyperpolarization shift in the conductance–voltage relation	[113,116,117]
	oocytes	interaction with SM. Hydrolysis of SM into ceramide-1-phosphate induces hyperpolarization shift in the conductance–voltage relation; hydrolysis of SM into ceramide decreases current to 90% and reduces gating currents	[113]
	oocytes	interacts with SM probably through the S3b and S4 voltage-sensing domains	[116]
TRPA1	rat trigeminal neurons	SM hydrolysis and inhibition of de novo synthesis of ceramide decrease AITC-induced Ca^2+^ uptake, which is not due to an increase in ceramide or sphingosine	[118]
	rat peripheral sensory nerve terminals	SM hydrolysis inhibits AITC-induced release of CGRP, which is not due to an increase in ceramide or sphingosine	[118]
TRPM8	rat trigeminal neurons	SM hydrolysis and inhibition of de novo synthesis of ceramide decrease icilin-induced Ca^2+^ uptake	[118]
TRPV1	rat trigeminal neurons	SM hydrolysis as well as inhibition of the synthesis of GSLs and de novo ceramide decrease both capsaicin- and resiniferatoxin-evoked Ca^2+^ uptake	[119]
	rat trigeminal neurons	SM hydrolysis and inhibition of de novo synthesis of ceramide decrease capsaicin-induced Ca^2+^ uptake, which is not due to an increase in ceramide or sphingosine	[118]
	rat peripheral sensory nerve terminals	SM hydrolysis inhibits capsaicin-induced release of CGRP, which is not due to an increase in ceramide or sphingosine	[118]
***GPCRs***			
AMPA receptor	rat hippocampal neurons	disruption of microdomains by simultaneous cholesterol depletion and CERS inhibition results in fewer, but larger receptor clusters; loss of synapses and dendritic spines	[82]
GABA_A_	rat hippocampal neurons	disruption of microdomains by simultaneous cholesterol depletion and CERS inhibition result in fewer, but larger receptor clusters, meaning reduced synapse number; loss of synapses and dendritic spines	[82]
NMDA receptor	rat forebrain	localized into PSD-95-rich microdomains and synaptic microdomains	[107]
	rat hippocampal neurons	generation of ceramide by TNFα-induced activation of nSMase2 stimulate NMDA receptor clustering	[102]
	CA1 pyramidal cells in rat hippocampal slices	C_2_-ceramide induces a sustained synaptic current depression probably mediated through the activation of protein phosphatases 1 and/or 2A	[120]
	rat hippocampal slices	long-term treatment with S1P agonist increases phosphorylation and membrane level of NMDA receptor subunit GluN2B probably through activation of the microdomain-associated Src kinase Fyn	[121]
serotonin_1A_ receptor	CHO cells	inhibition of ceramide synthesis leads to impaired function of the serotonin_1A_ receptor due to reduced ligand binding	[122]
serotonin_7_ receptor	HeLa cells	inhibition of ceramide and GSL synthesis reduces maximum agonist binding	[123]
***other receptors***			
Trk A	PC12 cells	GM1 directly associates with Trk and enhances neurite outgrowth and neurofilament expression induced by nerve growth factor (NGF)	[66]
		GM1 enhances NGF-dependent homodimerization of Trk	[68]
		GM1 depletion by inhibition of GluCer synthase inhibits NGF-induced neurite outgrowth, which is abolished by co-treatment with GM1	[67]
EGFR	mouse neural stem cells	GD3 mediates membrane microdomain localization of EGFR; ablation of GD3 results in reduced level of EGFR expression and accelerates EGF-induced EGFR degradation leading to decreased self-renewal capability	[59]
insulin receptor	*CerS2^−/−^* mouse liver	lack of C22–C24 ceramides inhibits phosphorylation and translocation of the insulin receptor into microdomains upon insulin stimulation	[124]
	Huh7 cells	clustering of GM2 inhibits signalling through the insulin receptor by excluding the receptor from non-caveolar membrane microdomains	[125]
	3T3-L1 adipocytes	TNFα-induced accumulation of GM3 eliminates insulin receptor from microdomains and inhibits insulin signalling	[126]
		GM3 disturbs interaction between the insulin receptor and caveola protein Cav-1 resulting in exclusion of the receptor from caveola and impairs insulin signalling	[127]
		inhibition of GluCer synthase counteracts TNFα-induced abnormalities in insulin signalling by normalizing GM2 and GM3 levels	[128]

The multifaceted nature of membrane microdomains is reflected in the way they regulate ion channel and receptor functions. The effect can be direct through protein–lipid interactions, but also more indirect by influencing the physical properties of the membrane. The consequence of the effect is highly dependent on the ion channel/receptor in question, and can include alterations in kinetics, membrane localization and trafficking. Yet some overall regulation strategies can be deduced, which are described in the following sections.

The list of sphingolipid-binding proteins is expanding, but only relatively few sphingolipid-binding motifs have been identified [129–131]. SM has been shown to regulate the activity of the Kv2.1 channel by interacting with the helix-turn-helix motif found in the S3b and S4 voltage-sensing domains of the channel in oocytes [113,116]. Hydrolysis of SM into ceramide profoundly inhibits K^+^ conductance along with ionic and gating currents [113]. The latter was also observed for the Kv1.3 channel pointing towards a general regulation mechanism of the channel's voltage sensor by SM. Furthermore, hydrolysis of SM into ceramide-1-phosphate causes a hyperpolarization shift in the conductance–voltage relation along with slowing of the deactivation, which overall leads to a stabilization of the open state of Kv2.1 [113,116,117]. However, removal of SM phospho-heads also inhibits K^+^ conductance of the Kir1.1 channel, which contains no voltage sensor, indicating that SM has several modes of ion channel regulation [113].

The major feature of microdomains is their ability to include or exclude proteins and thereby dictate which proteins are in close proximity to each other. The tightly packed microdomains favour incorporation of molecules with saturated and unbranched side chains, and thus many of the proteins that reside in the microdomains are often acylated, primarily palmitoylated and/or myristoylated [132,133]. Acylated proteins include postsynaptic density protein 95 (PSD-95), caveolin, GPI-anchored proteins, Src-family of tyrosine kinases and the neural protein GAP-43 [132,134]. As several ion channels are regulated by phosphorylation, co-localization of ion channels with kinases provides a convenient mode of ion channel modulation. It has been shown that Kv1.5 associates with the Src kinase Fyn in mammalian hippocampus through Kv1.5's Src homology 3 (SH3) domain [135]. This association facilitates phosphorylation of Kv1.2 and Kv1.4 subunits, which both lack the SH3 domain, but reside in a heteromultimeric complex with the Kv1.5 subunit. The phosphorylation of Kv1.2 and Kv1.4 leads to suppression of depolarization-evoked currents [135]. Kv1.5 is also an example of an ion channel that localizes to caveolin-rich microdomains. Interestingly, disruption of the microdomains by cholesterol depletion and hindering of ceramide synthesis by inhibition of CERS activity cause hyperpolarizing shifts in both the voltage-dependent activation and inactivation of Kv1.5 in Ltk cells [115].

The strategy of targeting ion channels/receptors to microdomains varies depending on the specific ion channel/receptor. Protein acylation is one strategy, as mentioned above, while recruitment to microdomains through binding to an acylated microdomain-residing protein has proven to be another strategy. PSD-95, a major synaptic scaffolding-protein, is an example of such a protein in the postsynaptic membrane. Palmitoylation of PSD-95, a PSD-95/Dlg/ZO-1 (PDZ) domain protein, localizes it to microdomains to which it has been shown to recruit the Kv1.4 ion channel and NMDA receptor subunits through interaction with the PDZ domain [134,136]. The recruitment of Kv1.4 is eliminated when palmitoylation of PSD-95 is prevented [134,137]. Interestingly, disturbance of the SM/ceramide balance by inhibition of nSMase results in increased level of PSD-95 in mice brain, which further leads to changes in NMDA subunit composition and an increase in AMPA receptors [103]. This illustrates the ripple effect that can occur when alterations in synaptic sphingolipids affect central synapse functions.

Sphingolipids have also proven to be important for the ability of receptors to bind and respond to ligands. Blockage of ceramide synthesis resulting in SM depletion leads to loss of agonist binding to the serotonin_1A_ and serotonin_7_ receptors in CHO cells and HeLa cells, respectively [122,123]. Furthermore, disturbance of microdomains has been shown to regulate initiation of signal transduction through nicotinic acetylcholine receptors (nAChRs). Simultaneous cholesterol removal and hydrolysis of SM into ceramide in rat hippocampal neurons increased the rate of recovery from desensitization and agonist affinity of the neuronal α7 nAChR, which overall led to slowing of the desensitization kinetics [112]. However, the same treatment gave an opposite effect for the α3β2 nAChR where the desensitization half-time was decreased [112]. This underlines the very individual nature of how ion channels are being regulated by microdomains.

Collectively, the mechanism by which ion channel/receptor functions is altered, and as a consequence of changes, microdomain composition remains elusive in most cases. There are many possible scenarios of how changes in the sphingolipid metabolism may affect the synaptic structure and hence function: lack of lipid–protein interaction, mislocalization, incorrect assembly of ion channel/receptor subunits, hindering of activity-regulating proteins/factors, changes in trafficking, altered agonist affinity and so on. Extensive research is needed in order to decipher the role of sphingolipids in regulation synaptic function through microdomains.

## 6. Sphingolipids and microdomains in neurological diseases

In the previous sections, we have discussed how alterations in sphingolipid metabolism can lead to abnormal organization and functions of membrane microdomains, and how functions of many neuronal ion channels and receptors depend on proper microdomain composition and integrity. Not surprisingly, defects in the sphingolipid metabolism have been linked to numerous neurological diseases, including Alzheimer's disease (AD), Parkinson's disease (PD), several types of epilepsy, Huntington's disease, Krabbe's disease, Gaucher's disease, inherited sensory and autonomic neuropathy, and dementia. This section outlines examples of how sphingolipids and microdomains are involved in the development of neurological diseases.

### 6.1. Alzheimer's disease

One of the major characteristics of AD is accumulation of the amyloid beta-peptide (Aβ), which ultimately leads to formation of plaques linked to disease progression. Several key enzymes associated with AD have been shown to localize to membrane microdomains including amyloid precursor protein (APP), β-site APP cleaving enzyme (BACE-1), γ-secretase complex and neprilysin (an Aβ-degrading enzyme) (reviewed in [2,18,138]). Co-localization of APP and secretases in microdomains promotes APP processing leading to accumulation of Aβ, which is abolished upon microdomain disturbance by cholesterol depletion [139]. An SM-binding motif has been identified in Aβ, and *in vitro* studies have shown that SM promotes aggregation of Aβ [130,140]. Accumulation of Aβ leads to SM depletion by activation of SMase, which is thought to disrupt a range of protein–lipid interactions and hence downstream signalling pathways [141]. Furthermore, activation of aSMase correlates with reported elevated levels of ceramide in the brain and cerebrospinal fluid of AD patients, which possibly is a result of increased expression of CERS1, CERS2, aSMase, nSMase and galactosylceramidase [142–146]. Spreading of plaque formation in the brain is thought to involve ceramide-enriched exosomes of Aβ and phosphorylated Tau [147]. A recent study has shown that ablation of nSMase in the AD mouse model 5xFAD improves AD pathology by reducing brain exosomes, ceramide levels, Aβ, phosphorylated Tau and plaques [148]. Thus, tilting the SM/ceramide balance towards ceramide contributes to the development of AD.

Evidence points towards gangliosides contributing to the initiation and progression of AD. Using model membranes, it has been shown that Aβ can bind to GM1 and that GM1 facilitates Aβ aggregation in membrane microdomains [149,150]. Consistently, increased levels of GM1 and GM2 have been found in microdomains isolated from the frontal and temporal cortex of AD patients and in brains of AD mice models, which correlate with accelerating plaque formation [151–153]. Recently, GM1 has been proposed to have a protective role towards Aβ aggregation rather than contributing to it. Using physiological concentrations of GM1 and Aβ in model membranes, it has been shown that GM1 in nanodomains does not induce Aβ oligomerization, but rather prevents SM-induced aggregation [140]. Thus, as the overall level of GM1 decreases during ageing [154], the protective role of GM1 decreases, thereby contributing to the onset of AD. However, it has been shown that GM1 is enriched in microdomains isolated from mice synaptosomes in an age-dependent manner despite an overall reduction of GM1 with age [57], indicating that regionally GM1 might facilitate plaque formation. The latter is supported by enrichment of GM1 and GM2 found in microdomains isolated from AD patients [151]. Additional studies are necessary in order to elucidate the role of GM1 in plaque formation in AD, which probably depends on the timing of disease onset [152].

It is evident that AD is accompanied by deregulated sphingolipid metabolism, yet the precise mechanisms behind AD pathogenesis need to be clarified. Meanwhile, a sphingolipid profile and microdomain composition might function as a diagnostic tool in the development of AD.

### 6.2. Parkinson's disease

The cause of PD is generally not known, but it is characterized by accumulation and fibrillation of α-synuclein in neurons leading to neurodegeneration. An increasing number of studies report that mutations in the glucocerebrosidase (GCase) gene confer increased susceptibility to the development of PD [155–158]. A reduced activity of GCase has been found in the brain of PD patients [159,160]. In line with this, GCase deficiency promotes accumulation of α-synuclein in cultured neurons [161]. GCase is located in lysosomes where it cleaves GluCer into ceramide and glucose. α-synuclein has been shown to bind to gangliosides, sharing the GluCer core structure, derived from the human brain [162]. GCase deficiency leading to increase in GluCer has been shown to control intracellular accumulation of α-synuclein in mice and human brains as well as in cultured neurons [161]. Additionally, the assemblies of α-synuclein were shown to inhibit normal activity of GCase and maturation of lysosomes, thereby contributing to pathology [161].

Other studies show no changes in the level of GluCer in human PD brains [163,164], indicating that GluCer is not pivotal to PD development. The attention has turned to membrane microdomains as α-synuclein has been observed to bind to lipids within these microdomains [165]. Indeed, membrane microdomains isolated from the frontal cortex of patients with incidental PD display profound alterations in lipid composition with a higher content of saturated lipids and lower content of unsaturated lipids as well as a reduction in cerebrosides and sulfatide, which overall indicates an increase in microdomain order [166]. GM1 has been of great interest as it binds α-synuclein, thereby promoting oligomerization. [167]. However, treatment of primate PD models with GM1 has shown beneficial effects including restoring neurochemical and physiological parameters [168–170]. Additionally, a study has shown that a consistent portion of PD patients have increased anti-GM1 antibodies [171]. The positive effect of GM1 may be explained by its ability to stabilize α-synuclein in an α-helix structure, thereby preventing fibrillation [167]. This effect is abolished in the familial PD mutant A30P of α-synuclein. Further studies are necessary in order to elucidate the role of membrane microdomains and sphingolipids in PD development.

### 6.3. Epilepsy

An increasing number of studies implicate defects in the sphingolipid metabolism, both in the biosynthesis and degradation pathway, with the development of epilepsy. Although our knowledge of how these defects affect membrane microdomains in the epileptic brains is limited, it can be speculated that the changed sphingolipid profiles perturb microdomain functions.

Recently, a homozygous mutation in the *CERS1* gene and a heterozygous deletion of the *CERS2* gene have been associated with the development of progressive myoclonic epilepsy [172,173]. CERS1 is the primary CERS in neurons responsible for synthesis of C18 ceramide. Downregulation of CERS1 in a neuroblastoma cell line induces ER stress and proapoptotic pathways, which points towards a role of CERS1 in neurodegeneration [172]. CERS1 deficiency in mice results in a pronounced decrease in brain gangliosides, along with diminution and neuronal apoptosis in the cerebellum [76,174]. Moreover, loss of CERS1 also causes impaired lysosomal degradation leading to accumulation of lipofuscin, which is a common mechanism observed in ageing and neurodegenerative diseases [76]. CERS1 deficiency in mice also leads to a reduction in MAG in oligodendrocytes, indicating how the lipid composition of neuronal membranes can affect the protein expression in oligodendrocytes [174]. CERS2 is the major CERS in oligodendrocytes, and lipidomic analysis of skin fibroblasts from the *CERS2^+/−^* patient shows that the SM profile resembles the changes in SM observed in the *CerS2^−/−^* mice [99,173,175,176]. CERS2 is important for maintaining membrane integrity shown by severely altered biophysical properties of membranes isolated from the brain of *CerS2^−/−^* mice [177]. Ablation of CERS2 in mice results in degeneration and detachment of myelin as well as cerebellar degeneration [99,175]. The latter again pinpoints the functional relationship between neurons and oligodendrocytes as insufficient myelination of neurons leads to their degeneration.

There have been multiple reports associating mutations in the gene encoding the aCDase with spinal muscular atrophy with progressive myoclonic epilepsy (SMA-PME) [178–181], although initially the interest of aCDase was on its involvement in the lysosomal storage disease Farber's disease [182]. Loss of aCDase in mice is embryonically lethal due to early apoptotic cell death [183]. It has been speculated that the development of SMA-PME instead of Farber's disease is a result of different residual activities of aCDase in the two diseases [182]. Knockdown of the aCDase orthologue in zebrafish compromises motor neuron axonal branching and increases apoptosis in the spinal cord [178]. It is known that increased levels of ceramide rearrange microdomains into larger membrane domains of which one of the possible outcomes is apoptosis [182,184]. Thus, control of ceramide levels is crucial in order to prevent neuronal loss.

Defect ganglioside biosynthesis has been associated with the development of epilepsy through the discovery of a homozygous loss-of-function mutation of the GM3 synthase gene linked to infantile-onset symptomatic epilepsy syndrome and refractory epilepsy [185,186]. Loss of GM3 synthase activity in the affected children was accompanied by complete lack of GM3 and its downstream biosynthetic derivatives in plasma with evidence of increased flux through the remaining functional ganglioside synthesis pathways [185]. However, a compensatory effect is not observed in patient-derived GM3 synthase-deficient skin fibroblasts, which have a 93% reduction in ganglioside content compared with control skin fibroblasts [187]. This leads to a decrease in EGF-induced proliferation as well as migration of the patient skin fibroblasts caused by lack of GM3 facilitation of EGF binding to the EGFR receptor, which is known to localize to membrane microdomains [187,188]. GM3 synthase-deficient mice show no obvious neurological defects [189], and thus an alternative model system must be employed in order to evaluate the role of GM3 synthase in brain membrane microdomains.

## 7. Concluding remarks

Genetically engineered mice models with defective sphingolipid metabolism at various stages of the sphingolipid pathway have paved the way for understanding how sphingolipids are involved in regulating the nervous system. The phenotypes observed in KO mice deficient in ganglioside synthases have often been milder than expected, pointing towards a redundancy in the functions of gangliosides. Yet some ganglioside functions are highly specific and cannot be substituted for by others. It is important to take into account that what we see in mice models might not be representative for humans. For instance, KO of the GM3 synthase in mice does not show any major abnormalities [190], while the human equivalent has been diagnosed with infantile-onset symptomatic epilepsy [185,186]. Thus, even though our knowledge of how the brain functions has expanded substantially through animal models, we must always keep in mind the limitations of these models.

Membrane microdomains play a central role in brain development and maintenance. The existence of membrane microdomains has been highly debated, but accumulating evidence indicates that the lipid composition of the plasma membrane is very heterogeneous and laterally organized into microdomains [191]. Technological advances such as stimulated emission depletion (STED) microscopy now allow us to visualize these former enigmatic compartments in living cells [16,17]. Perturbations of the sphingolipid metabolism affect dynamics, integrity and functions of the microdomains. Disarrangement of sphingolipid microdomains has been associated with numerous neurological diseases, and it has been proposed that analysis of membrane microdomain disorder can function as a diagnostic tool in the early diagnosis of neuropathological development [18]. The challenge is how to take advantage of this early diagnosis in the treatment of patients as it can be challenging to distinguish between primary and secondary effects. Future research will help clarify the role of sphingolipids in neurological disorders, and further reveal whether individual sphingolipid species or collective changes in the sphingolipid profile are primary effectors. This will be pivotal in the development of therapeutic strategies in treatment of sphingolipid related neurological diseases.
